# Alignment Before Apology: Transactional and Relational Dimensions of Reconciliation in Canadian Healthcare

**DOI:** 10.1177/08404704261471471

**Published:** 2026-08-07

**Authors:** Alika T. Lafontaine, Paula Cashin, Santanna Hernandez, Chris J. Lafontaine

**Affiliations:** 1 Department of Anesthesiology & Pain Medicine, Faculty of Medicine and Dentistry, 3158 University of Alberta, Edmonton, Alberta, Canada; 2 2129 Independent Researcher, Ottawa, Ontario, Canada; 3 2129 Department of Family Medicine, University of Calgary, Calgary, Alberta, Canada; 4 Formerly of Saulteaux Pelly Agency Health Alliance, Regina, Saskatchewan, Canada

## Abstract

The purpose of this article is to introduce the Social Dynamics of Change (SDoC), a framework for institutional transformation, and examine the Canadian Medical Association’s (CMA) 2024 Apology to the Indigenous Peoples of Canada as a case study in institutional reconciliation. Converging social, political, and institutional forces exposed the CMA’s maladaptive relationship with Indigenous Peoples. Through the SDoC framework, reconciliation involved movement from crisis through denial, resistance, exploration, and commitment. Progress required relational accountability, trust, co-design, and sustained engagement between Indigenous and non-Indigenous leaders. The case highlights the importance of Indigenous leadership in senior governance, as three authors in distinct leadership roles helped create the enabling environment that supported the CMA’s reconciliation journey. The CMA case suggests that meaningful institutional reconciliation is achieved when relational alignment precedes transactional milestones. The SDoC framework helps explain how healthcare organizations navigate change and translate reconciliation commitments into lasting shifts in governance and culture.

## Introduction

The Social Dynamics of Change (SDoC) extends the alignment model introduced by Lafontaine & Lafontaine in their 2019 retrospective analysis on reconciliation.^1^ In that paper, the authors argue that decades of dysalignment between Indigenous communities and healthcare institutions would ultimately require new pathways to achieve alignment. That warning proved prescient. Between 2020 and 2023, a convergence of social and political pressures transformed reconciliation from a stated aspiration within Canadian healthcare into an operational necessity.

The alignment framework conceptualized dysfunction in Indigenous health engagement as an incompatibility of priorities that were sustained by structures that bypassed relational accountability. While that model described the conditions that produce dysalignment, it did not fully articulate the process that explains how organizations move from alignment to transformation.

The SDoC is presented here as an original framework, developed by the first and fourth authors. The framework is examined through a case study of the Canadian Medical Association’s Apology to the Indigenous Peoples of Canada that was delivered in September 2024. The case is told from the inside by three of the authors who occupied distinct governance roles at the Canadian Medical Association (CMA) throughout the process. The first author served as CMA President-elect, President, and Past-President. The second author was the first Indigenous Board Director and Vice-Chair of the CMA Board of Directors. The third author was the first Indigenous medical student to serve on the CMA Board of Directors.

Each distinct role came with different forms of authority and access. These perspectives are analytically significant. Reconciliation within institutions is rarely determined by policy alone. It is shaped by who occupies leadership positions, the relationships those roles make possible, and the capacity of those relationships to sustain pressure over time. A central question is the distinction between transactional and relational work, and how to hold both at once.

## The Social Dynamics of Change

The Social Dynamics of Change is a framework for understanding institutional transformation and its underlying dynamics. The SDoC describes institutional transformation as a repeating sequence of phases: Status Quo → Crisis → Denial → Resistance → Exploration → Commitment → New Status Quo (Figure 1). The phase labels echo earlier stage models of change, notably the Kübler-Ross change curve^2^ and the Satir change model,^3^ but SDoC departs from them by redefining denial and resistance not as emotional stages of coping, but as distinct cognitive and behavioural mechanisms: denial as the persistence of incumbent mental models, and resistance as a material contest over the redistribution of resources. The vertical axis of the SDoC represents sentiment toward the status quo; the upper zone (“Let Go”) reflects openness to change and the release of established arrangements; and the lower zone (“Hold On”) reflects entrenchment and resistance to change.

**Figure 1.**
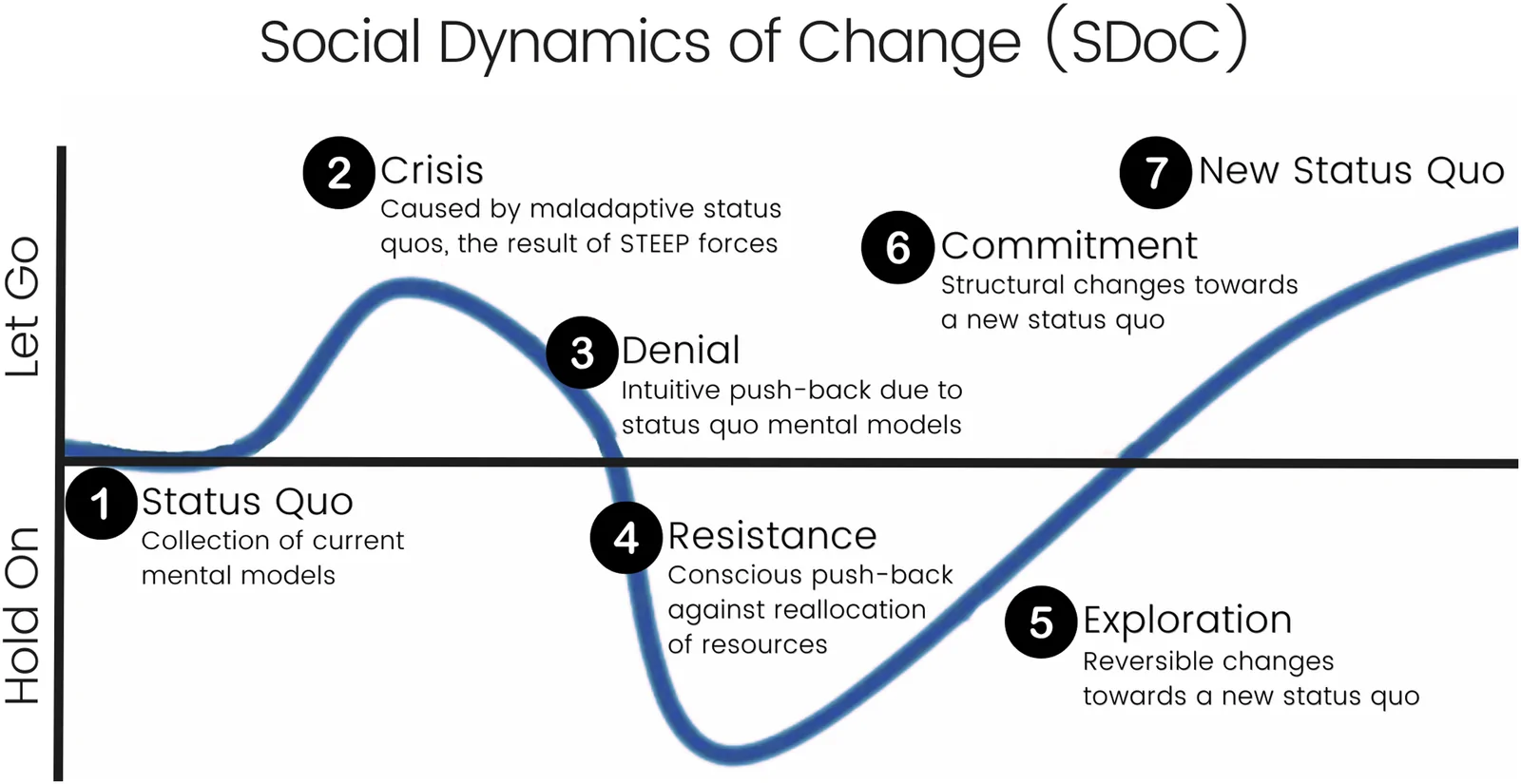
The Social Dynamics of Change (SDoC). Institutional transformation is shown as a repeating cycle of seven phases, plotted against sentiment toward the status quo, from entrenchment (“Hold On”) to openness (“Let Go”). Denial and Resistance are the two stall points at which most change fails and returns to the status quo. Movement through exploration and commitment establishes a new status quo, after which the cycle repeats

The cycle begins with the S*tatus Quo*: a stable collection of mental models and resource allocation that sustains existing priorities and power structures. Status quo remains unquestioned until it becomes maladaptive, typically when the operating environment changes faster than the institution’s ability to adjust and respond. Said simply, existing status quos no longer deliver desired results and grow increasingly dysaligned with the priorities of the stakeholders they serve. These external forces are described by STEEP, a variant of the environmental-scanning frameworks introduced by Aguilar^4^: social, technological, economic, environmental, and cultural/political pressures usually beyond a system’s ability to influence and control. When dysaligned status quos reach a certain point, crisis results.

*Crisis* is when dysalignment passes a threshold where stakeholders begin to question whether the status quo is effective. Crisis is where momentum for change is greatest. It is where the consequences of dysalignment surface: pain, suffering, injustice, eroded trust, and wasted resources. Left unaddressed, these compound, and dysalignment feeds crises that grow more frequent and severe which in turn, drive greater openness to changing the status quo. In the SDoC, crisis is invariably followed by two dynamics that work against institutional transformation, denial and resistance.

*Denial* is the first stall point in the change process. It is the persistence of existing mental models in the face of evidence that the models are maladaptive. Denial is a predictable cognitive and institutional response to disruption and uncertainty. Overcoming denial requires safe spaces where mental models can be surfaced without triggering defensiveness, and brave spaces where they can be critically examined and revised.^5^ Within the SDoC framework, co-design rather than confrontation is the method to advance change. Momentum without relational grounding risks future backlash that returns the institution to the status quo.

*Resistance* is the second and more deeply entrenched stall point. Whereas denial is primarily cognitive, resistance is material. It arises from concerns about what people and institutions stand to lose as the status quo shifts, including reputation, influence, financial resources, social relationships, and identity. Treating these actual or perceived losses as illegitimate will foreclose negotiation and slow the change process. Resistance must be engaged rather than overridden. The resistance stage is where most institutional change fails because resistance is mistaken for denial and treated as a problem requiring persuasion rather than a material problem that requires acknowledgment and negotiation of loss.

*Exploration* marks the turning point at which an organization moves beyond the stall points and there is a genuine willingness to consider the requirements of a new arrangement. These changes remain reversible.

*Commitment* is reached when exploration results in durable structural changes, such as policy revisions, revised resource allocations, and governance shifts. A new status quo forms, and over time the cycle begins again as new status quos become maladaptive due to shifting STEEP forces.

## Case Study of the CMA Apology to the Indigenous Peoples of Canada

### Crisis: The STEEP Environment

The CMA’s historical relationship with Indigenous Peoples was characterized by dysalignment, a persistent incompatibility between Indigenous and institutional priorities. It was sustained by institutional policies and governance structures that excluded Indigenous participation in decision-making. For most of the CMA’s history, systemic racism in healthcare was treated as a contested claim rather than a documented fact.

STEEP forces had been pressing on that status quo for decades. The Oka Crisis and the subsequent Royal Commission on Aboriginal Peoples^6^ in the 1990s led to a CMA Working Group on Aboriginal Health and a submission to RCAP, but those efforts ebbed as the crisis appeared to wane. A more sustained reckoning built through the Indian Residential School Survivors Settlement, the Truth and Reconciliation Commission,^7^ the adoption of the United Nations Declaration on the Rights of Indigenous Peoples,^8^ and the National Inquiry into Missing and Murdered Indigenous Women and Girls,^9^ amplified by social media that made institutional silence visible and its consequences immediate. The COVID-19 pandemic brought gaps in healthcare access front-of-mind for most Canadians. By 2020, systemic racism was no longer debatable.^10^ It was documented, named in the In Plain Sight report^11^ and the coroner’s findings into the death of Joyce Echaquan.^12^ A posture built for a world where the existence of harm was disputed had become maladaptive in a world where that harm was formally recognized. Cumulatively, these forces exposed the CMA’s dysalignment with societal priorities and a shifting balance of power.

The CMA’s response lagged these forces and unfolded iteratively. Among them, the Truth and Reconciliation Commission’s 2015 final report was the catalyst: it named reconciliation in healthcare as a priority, and the CMA responded by making Indigenous health a strategic priority within its Impact 2040 strategy. It held its first reconciliation event at its 2016 annual meeting and expressed public and private support for reconciliation initiatives from 2017 onward. Internal work began in earnest in 2022, when the CMA established an Indigenous Guiding Circle (IGC) of Elders, Knowledge-Keepers, and Indigenous experts to guide its approach. In September 2022, the IGC proposed a formal apology as a priority. The CMA committed publicly in June 2023 and delivered the apology on September 18, 2024.^13^

In the background, active work to guide the momentum of crisis through denial and resistance was occurring across the institution.^14^ For the first time in the CMA’s history, the Board of Directors included three Indigenous physicians, including the first Indigenous President, the first Indigenous Board Director and Vice-Chair, and the first Indigenous medical student Board Director. The three authors occupied these roles. What was happening externally to the CMA was suddenly being felt internally, with first-person lived experience being communicated directly to its Board and staff.

The IGC and Indigenous representation amplified the crisis further, pushing the CMA into a binary choice. It could maintain its status quo and become increasingly maladaptive, forfeiting both its opportunity to positively impact the lives of Indigenous Peoples and its standing as a national leader in healthcare, or begin to let go of existing assumptions and explore a different future for the institution.

### Denial: Co-Designing New Mental Models

Denial took recognizable forms within the CMA. As an institution nearly as old as Canada itself, the mental models sustaining it were not necessarily expressions of malice. They were the accumulated logic of a Canadian institutional culture that had long understood medicine’s relationship with Indigenous Peoples as a matter of individual physician conduct rather than collective institutional responsibility. This same logic understood the gesture of an apology as distinct from its substance and the authoring of an apology as something that should be managed by the institution rather than co-developed with those who had experienced harm.

Moving through denial required creating conditions under which these mental models could be accurately named and honestly examined. Safe spaces, where existing perspectives could be articulated without triggering defensiveness, were as necessary as brave spaces in which those perspectives could be interrogated and revised, spaces which the three authors resided in throughout the process.

The momentum generated to overcome denial carried inherent risks. Managing the pace of change and sustaining the relational infrastructure capable of absorbing institutional pressure were as consequential as any formal decision made during this period. Denial cannot be confronted out of existence; it must be replaced through the development of new cognitive and cultural understandings.

### Resistance: Negotiating Redistribution

Resistance within the CMA process concerned resources, concrete ones. What was at stake included reputational standing for organizations, physicians, and leaders who had not previously understood themselves to be implicated in systemic harm; voice, meaning whose perspectives would shape the content and scope of the apology; financial resources redirected toward Indigenous health priorities; social relationships within the medical establishment that might be disrupted by the work; and identity, including what it means to be a physician, a healer, an institution committed to the principle of first doing no harm. Within the SDoC framework, these losses were not symbolic. Treating them as such would have foreclosed the negotiation before it began.

What made negotiation possible was the quality of the relationships built long before resistance emerged. Through years of engagement within the CMA, the three authors had accumulated a form of relational capital that is rarely named in institutional change literature, yet is foundational to transformation. Through iterative friction, including disagreements navigated with honesty and difficult conversations held without rupture, they developed trusting relationships with the Chief Executive Officer, executive leadership, staff, board members, and the Board Chair. These relationships had been tested over time and had endured. As a result, the choices available at the most difficult moments were shaped by what had been built during the quieter ones.

At several pivotal moments, the work became a negotiation among three sources of voice: Indigenous voices within the CMA, non-Indigenous voices within the CMA, and both Indigenous and non-Indigenous external voices. These groups held different relationships to the apology and different understandings of what meaningful reconciliation required. Their perspectives did not always align.

Some external stakeholders sought to move authority over the apology’s content and direction from the CMA’s internal process to external advisors, framing this in the language of self-determination, lived experience, and expertise. It carried real force, since reputational standing in reconciliation work is itself a resource. The CMA thus faced two legitimate but unequal forms of authority: external advisory authority grounded in reputational and moral standing and internal fiduciary authority grounded in governance accountability. The second author engaged this directly, distinguishing not between Indigenous voices but between accountability structures. External advisors bring expertise, but Board members remain accountable for the decisions and their consequences. The question was not whose voice should prevail, but how to balance forms of authority within a process that required both accountability and legitimacy.

The decision to stand behind internal Indigenous leadership was not a rejection of external Indigenous expertise. The Indigenous Guiding Circle and Elders who were woven throughout the process provided relational space and cultural authenticity that internal leaders alone could not generate. Similarly, CMA staff bridging institutional and Indigenous worlds played a critical role in cultivating relationships and sustaining the work over time. What the organization chose was clarity regarding accountability. Those with fiduciary responsibility for the CMA, and who had built relationships from within the organization, would remain accountable for the direction and outcomes of the process.

This was possible because the Board of Directors was willing to follow, not because the destination was certain (it was not), but because the relationships between the three authors and the Board of Directors had been built through sufficient iterative friction to generate trust in conditions of uncertainty.

### Exploration and Commitment

At the time of the apology, the CMA had progressed through denial and resistance and entered a phase of exploration that was characterized by a genuine willingness to examine what reconciliation required in practice. Two years after the apology, there is evidence of a transition into the commitment phase with revisions to the Code of Ethics and significant funding directed to Indigenous health priorities. These actions suggest that alignment is becoming embedded into institutional structures, governance, and resource allocation, rather than remaining solely a matter of language or intention.

## Implications for Health Leadership and Institutional Reconciliation

The case yields four lessons for institutional reconciliation beyond the CMA. Transactional milestones such as apologies and policy revisions are necessary but insufficient; without relational accountability they become the ceiling of reconciliation rather than its foundation. Denial cannot be confronted out of existence; it must be replaced through co-design in safe and brave spaces. Resistance names real losses of reputation, voice, resources, relationships, and identity, and the path through it is negotiation, not persuasion. Above all, reconciliation is a design process with identifiable phases, and the core leadership competency is recognizing which phase an organization occupies and applying the strategy that fits it.

## Conclusion

The CMA Apology to the Indigenous Peoples of Canada was not the destination. It was evidence that the SDoC cycle can progress from crisis through denial and resistance towards exploration and commitment. Viewed through the SDoC framework, the CMA Apology represents a transactional milestone that emerged from a meaningful relational process. It suggests that institutional transformation may depend less on policies than on the enabling environment that makes change possible and the relationships within them.

Creating and sustaining that enabling environment requires leaders who can hold relational space under pressure, relationships strong enough to withstand challenge, and organizations willing to move forward when the path ahead is uncertain. The CMA case further highlights the importance of Indigenous leadership in senior governance. Indigenous leaders help create the conditions necessary to sustain momentum, build trust, and maintain accountability throughout the process.

## Data Availability

Data sharing is not applicable to this article, as no datasets were generated or analyzed.*
